# p38 mediates mechanical allodynia in a mouse model of type 2 diabetes

**DOI:** 10.1186/1744-8069-6-28

**Published:** 2010-05-19

**Authors:** Hsinlin T Cheng, Jacqueline R Dauch, Sang Su Oh, John M Hayes, Yu Hong, Eva L Feldman

**Affiliations:** 1 Department of Neurology, University of Michigan Medical Center, Ann Arbor, Michigan, USA

## Abstract

**Background:**

Painful Diabetic Neuropathy (PDN) affects more than 25% of patients with type 2 diabetes; however, the pathogenesis remains unclear due to lack of knowledge of the molecular mechanisms leading to PDN. In our current study, we use an animal model of type 2 diabetes in order to understand the roles of p38 in PDN. Previously, we have demonstrated that the C57BLK db/db (db/db) mouse, a model of type 2 diabetes that carries the loss-of-function leptin receptor mutant, develops mechanical allodynia in the hind paws during the early stage (6-12 wk of age) of diabetes. Using this timeline of PDN, we can investigate the signaling mechanisms underlying mechanical allodynia in the db/db mouse.

**Results:**

We studied the role of p38 in lumbar dorsal root ganglia (LDRG) during the development of mechanical allodynia in db/db mice. p38 phosphorylation was detected by immunoblots at the early stage of mechanical allodynia in LDRG of diabetic mice. Phosphorylated p38 (pp38) immunoreactivity was detected mostly in the small- to medium-sized LDRG neurons during the time period of mechanical allodynia. Treatment with an antibody against nerve growth factor (NGF) significantly inhibited p38 phosphorylation in LDRG of diabetic mice. In addition, we detected higher levels of inflammatory mediators, including cyclooxygenase (COX) 2, inducible nitric oxide synthases (iNOS), and tumor necrosis factor (TNF)-α in LDRG neurons of db/db mice compared to non-diabetic db+ mice. Intrathecal delivery of SB203580, a p38 inhibitor, significantly inhibited the development of mechanical allodynia and the upregulation of COX2, iNOS and TNF-α.

**Conclusions:**

Our findings suggest that NGF activated-p38 phosphorylation mediates mechanical allodynia in the db/db mouse by upregulation of multiple inflammatory mediators in LDRG.

## Background

Diabetic neuropathy affects up to 50% of patients with either type 1 or type 2 diabetes [1,2]. Among the multiple symptoms of diabetic neuropathy, painful diabetic neuropathy (PDN) is the most devastating complication of diabetes [3]. Although there are multiple presentations of PDN in diabetic patients, the most common symptoms of PDN result from a length-dependent polyneuropathy starting at the longest axonal terminals in the feet and extending toward the body [3]. The upper extremities become affected in a similar fashion as the disease progresses, eventually forming the characteristic glove and stocking distribution of sensory symptoms [1,2]. It is believed that PDN is an early component of diabetic neuropathy [4,5]. PDN is detected more frequently in patients with prediabetic condition or impaired glucose tolerance than in patients with late-stage diabetes [5]. Similar to other types of neuropathic pain, PDN has features of allodynia and hyperalgesia [6]. Allodynia is defined as increased nociceptive perception to normally innocuous stimuli and hyperalgesia indicates increased nociception to normally painful stimuli. Although PDN is a common symptom among diabetic patients, its mechanisms remain unclear. Current treatments for PDN are not effective and less than 30% of patients obtain satisfactory pain relief [6].

Mitogen activated protein kinases (MAPK) are a group of intracellular messenger proteins that transmit signals from cell membrane receptors to the nucleus. The MAPK family consists of extracellular signal-regulated protein kinases (ERKs), p38, and c-Jun N-terminal kinase (JNK). All MAPKs are involved in both inflammatory and neuropathic pain [7,8]. However, individual MAPKs could play distinct roles in different pain models [8]. p38 is a serine-threonine kinase which is activated by phosphorylation and mediates many cellular responses to a variety of chemical and physical insults [9]. In a model of NGF-induced hyperalgesia, p38 is phosphorylated in Trk A-positive small to medium-sized dorsal root ganglian (DRG) neurons and phosphorylated p38 (pp38) mediates NGF-induced upregulation of nociceptive molecules [10]. In addition, peripheral inflammation and axotomy also activate p38 in both DRG neurons and spinal cord microglia [11-13]. Administration of a p38 inhibitor, SB203580, reverses p38-mediated pain in several pain models [9,14]. Inhibition of p38 activation prevents the development of PDN in streptozotocin (STZ)-treated animal models of type 1 diabetes [14].

PDN is more prevalent in type 2 than type 1 diabetes [6], yet most published studies for PDN use animal models of type 1 diabetes. Understanding the mechanisms of PDN as it develops in the context of type 2 diabetes could lead to developments of effective treatments to target this devastating disease. We have previously characterized the db/db mouse as a model for PDN of type 2 diabetes [15]. The db/db mouse carries a homozygous null mutation of the leptin receptor [16,17]. We reported that db/db mice develop features of PDN, including mechanical allodynia at 6-12 wk of age and evident sensory neuropathy at 24 wk of age [15,17]. We reported that the mechanical allodynia in db/db mice is concordantly associated increased NGF/Trk A receptor signaling in DRG neurons. In the current study, we examine the roles of p38 in the development of mechanical allodynia in db/db mice. We hypothesized that NGF-Trk A signaling could trigger p38 activation and upregulate p38-depedent nociceptive molecules in LDRG of db/db mice. Our findings provide new understanding of the molecular mechanisms of PDN of type 2 diabetes and indicate that p38 could be a potential target for treating PDN of type 2 diabetes.

## Results

### p38 is phosphorylated during the time period of mechanical allodynia

**Figure 1:**
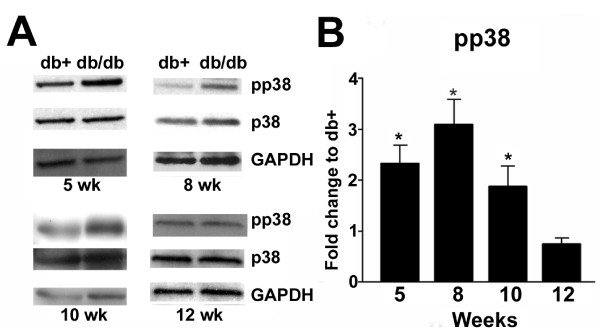
**Phosphorylation of p38 during the course of mechanical allodynia**. A: Representative immunoblots of pp38, p38 and GAPDH using LDRG extracts from db+ and db/db mice at 5, 8, 10, and 12 wk of age. Increased levels of pp38 were detected in db/db mice when compared to db+ mice at 5, 8 and 10 wk of age. At 12 wk of age, p38 phosphorylation returned to the control level. In addition, no change in expression levels of p38 was detected in LDRG of db/db mice and db+ mice. GAPDH served as the loading control. B: Densitometric analysis of immunoblots using LDRG of db+ and db/db mice at 5, 8, 10 and 12 wk of age during the development of mechanical allodynia. Significantly enhanced pp38 levels were detected in db/db mice at 5, 8 and 10 wk of age but not at 12 wk of age. N = 4, *p < 0.05.

We previously detected the development of mechanical allodynia in db/db mice from 6-12 wk of age. The maximum reduction of mechanical pain thresholds was detected at 8 wk of age [15]. In the current study, pp38 immunoblots were performed on LDRG collected at 5, 8, 10, and 12 wk of age (Fig. 1). Representative pp38 immunoblots demonstrated increased phosphorylation of p38 in LDRG of the db/db mouse model in comparison to the db+ mouse model at 5, 8 and 10 wk of age. At 12 wk of age, p38 phosphorylation in db/db mice was reduced to the control level (Fig. 1A). There was no difference in levels of total p38 protein expression between db+ and db/db mice. Densitometric studies which normalized the levels of pp38 to that of total p38 and actin revealed significantly increased p38 phosphorylation in the db/db mouse at 5, 8, and 10 wk of age. No significant difference between the pp38 levels of db+ and db/db mice was detected at 12 wk of age (Fig. 1B).

### pp38 immunoreactivity is detected in small- to medium-sized LDRG neurons during the time period of mechanical allodynia

**Figure 2:**
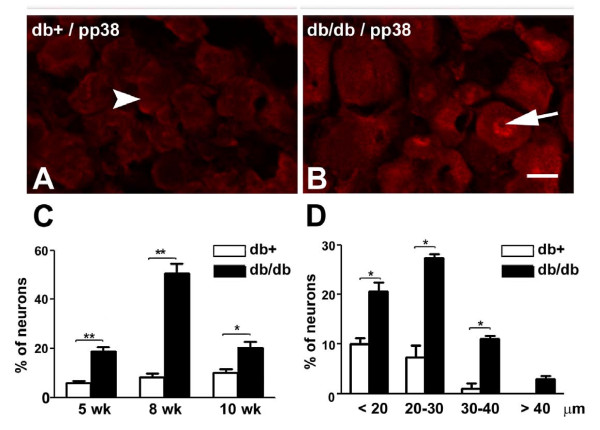
**pp38 immunohistochemistry in LDRG of db+ and db/db mice**. A, B: Representative pictures of pp38 immunohistochemistry in DRG from db+ (A) and db/db (B) mice at 8 wk of age. Increased nuclear immunoreactivity was detected in db/db (arrow) but not db+ (arrowhead) LDRG neurons. Bar = 15 μm. C: Quantitation of pp38-positive LDRG neurons in db+ and db/db mice. Significantly increased numbers of pp38-positive LDRG neurons were detected in db/db mice in comparison with db+ mice. N = 6, *p < 0.05, **p < 0.01. At 8 wk of age, the maximum increase in the percentage of pp38-positive neurons was detected in comparison to 5 and 10 wk LDRG. *p < 0.05, one-way ANOVA test. D: Cell size distribution of pp38-positive LDRG neurons at 8 wk of age. Significant increase of pp38-positive LDRG neurons were detected in neurons <20 μm, 20-30 μm and 30-40 μm in diameter. Small numbers of large DRG neurons (<40 μm) were also positive for pp38 in db/db mice but not db+ mice. N = 4, *p < 0.05.

We next localized pp38 in L4-6 DRG using immunohistochemistry (Fig. 2). Only trace amounts of pp38 immunoreactivity were detected in L4-6 DRG of db+ mice (Fig. 2A, arrowhead). In L4-6 DRG of db/db mice, pp38 immunoreactivity was detected mostly in the nuclei of small- to medium-sized neurons (Fig. 2B, arrow). Quantification studies demonstrated that there is a significant increase in the percentage of pp38-positive neurons in db/db mice compared to db+ mice of the same age during the period of mechanical allodynia (Fig. 2C). The most prominent increase was detected at 8 wk of age, with a 6-fold increase in the percentage of pp38-positive neurons in db/db mice in comparison to db+ mice. A cell size distribution study detected significant increases of not only the percentages of pp38 positivity in small- (<20 μm in diameter) to medium-sized (20-40 μm in diameter) LDRG neurons, but also in large-sized (>40 μm in diameter) LDRG neurons in db/db mice at 8 wk of age (Fig. 2D).

### Anti-NGF inhibits the phosphorylation of p38 in db/db mice

**Figure 3:**
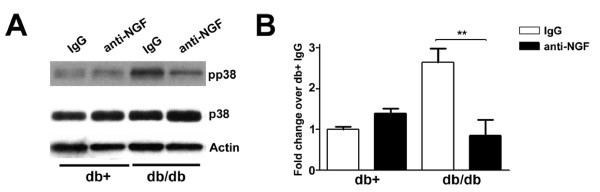
**Anti-NGF treatment inhibits the phosphorylation of p38 in db/db mice**. A: Representative immunoblots of pp38, p38 and actin using LDRG from db+ and db/db mice treated with either control (IgG) or an anti-NGF antibody for 2 wk. Anti-NGF treatment decreased the intensity of pp38 signals in db/db mice but not db+ mice at 8 wk of age. B: Densitometric studies of pp38 immunoblots using LDRG from 8 wk old db+ and db/db mice. A significant decrease in p38 phosphorylation was detected in db/db mice after anti-NGF treatment. N = 4, **p < 0.01

Previously, we reported enhanced NGF expression and phosphorylation of Trk A receptors in LDRG of db/db mice during the period of mechanical allodynia. The time course of NGF-Trk A signaling parallels the current findings of p38 phosphorylation [15]. To investigate if NGF is an upstream activator of p38 during the period of mechanical allodynia in db/db mice, we administered the same anti-NGF antibody used in our previous studies to block the development of mechanical allodynia [15]. Pp38 immunoblots were performed to determine the effects of anti-NGF on p38 phosphorylation in LDRG following 2 weeks of anti-NGF treatment, following the same paradigm as our previous studies [15]. Treatments with anti-NGF at 6 and 7 wk of age significantly decreased the phosphorylation of p38 in LDRG of db/db mice at 8 wk of age (Fig. 3A). Densitometric studies further determined that anti-NGF reversed the p38 phosphorylation in db/db mice but did not affect the baseline p38 phosphorylation in LDRG of db+ nondiabetic mice (Fig. 3B).

### Intrathecal administration of SB203580 inhibits p38 phosphorylation and mechanical allodynia

**Figure 4:**
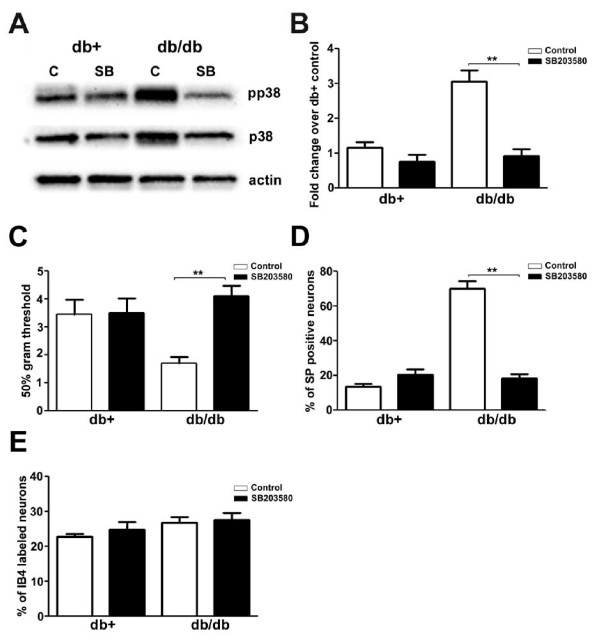
**SB203580 inhibits p38 phosphorylation, mechanical allodynia, and the percentage of SP-positive LDRG neurons in db/db mice**. A: Representative immunoblots of pp38, p38 and actin from db+ and db/db LDRG at 8 wk of age after 1 wk of intrathecal treatments of vehicle (control CSF with 10% DMSO) (C) or vehicle with SB203580. SB203580 treatment decreased levels of p38 phosphorylation in db/db mice but not in db+ mice. B: Densitometric studies of pp38 immunoblots demonstrated significant inhibition of p38 phosphorylation in db/db mice. C: SB203580 treatment reversed the decrease in mechanical thresholds (allodynia) in db/db mice. D: SB203580 treatment also lowered the elevated percentages of SP-positive LDRG neurons in control db/db mice compared to control db+ mice. E: SB203580 treatment had no effect on the percentage of IB4-labelled LDRG neurons in db+ and db/db mice. N = 4, **p < 0.01.

To determine directly if the activation of p38 mediates mechanical allodynia in db/db mice, a p38 inhibitor, SB203580, was administered intrathecally via mini osmotic pumps. Minipumps were inserted into both db+ and db/db mice at 7 wk of age and delivered SB203580 in artificial CSF with 10% DMSO at a rate of 0.51 μg/hr for 7 d. Control groups were treated with artificial CSF with 10% DMSO. Mechanical thresholds were measured at the end of the SB203580 treatment. pp38 immunoblots confirmed the inhibition of p38 phosphorylation in LDRG in response to SB203580 treatment in both db+ and db/db mice (Fig. 4A). Densitometric analysis demonstrated a reversal of p38 phosphorylation in db/db mice down to the levels observed in the control db+ mice (Fig. 4B). In conjunction with the inhibition of p38 phosphorylation, SB203580 treatment inhibited mechanical allodynia in db/db mice (Fig. 4C) and lowered the percentage of substance P (SP)-positive neurons (Fig. 4D). In contrast, the percentage of Isolectin B-4 (IB4)-labeled neurons was not affected by SB203580 treatment (Fig. 4E).

### Intrathecal administration of SB203580 inhibits the upregulation of inflammatory mediators in LDRG of db/db mice

**Figure 5:**
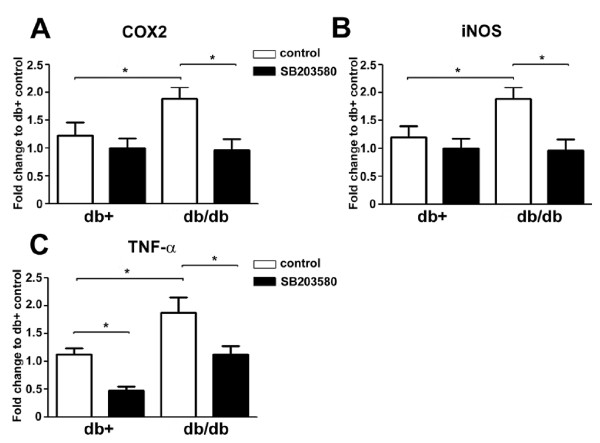
**SB203580 inhibits the upregulation of COX2, iNOS and TNF-α gene expression**. RT-PCR analysis of COX2 (A), iNOS (B) and TNF-α (C) in LDRG of db+ and db/db mice. The gene expression of all three inflammatory mediators was upregulated in LDRG of db/db mice. SB203580 treatment significantly decreased the elevated levels of gene expressions in db/db mice. In addition, SB203580 treatment lowered the gene expression of TNF-α in db+ mice. N = 4, *p < 0.05.

To study the downstream molecules of NGF-p38 signaling, we screened several nociceptive molecules reported to be involved in p38-mediated pain, including COX2, iNOS, and TNF-α. We first performed RT-PCR to measure the gene expression of these molecules (Fig. 5). Similar patterns of gene regulation were detected for all three proteins. The gene expression of COX2 (Fig. 5A), iNOS (Fig. 5B), and TNF-α (Fig. 5C) were all upregulated in LDRG of db/db mice in comparison to the db+ control. SB203580 treatment significantly decreased the upregulation of all three genes (Fig. 5A-C). SB203580 treatment did not affect the gene expression of these molecules in LDRG of db+ mice except in the case of TNF-α (Fig. 5C).

**Figure 6:**
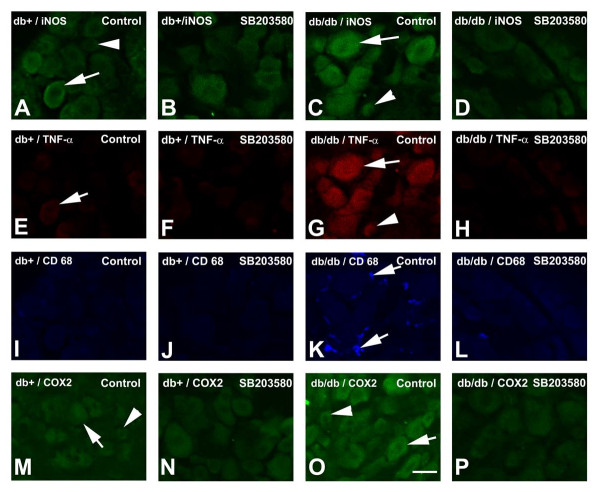
**Immunolocalization of iNOS, TNF-α, CD68, and COX2**. Immunohistochemistry studies were performed on LDRG of db+ (A, B, E, F, I, J, M, N ) and db/db mice (C, D, G, H, K, L, O, P). The mice were treated with vehicle control (A, C, E, G, I, K, M, O) or vehicle with SB203580 (B, D, F, H, J, L, N, P). In vehicle control treated mice, an increased percentage of iNOS-positive DRG neurons were detected in both large (arrow) and small to medium (arrowhead)-sized DRG neurons in db/db (C) in comparison with db+ (A) mice. Double immunofluorescent studies demonstrated that TNF-α immunoreactivity was detected in the iNOS-positive neurons of db/db mice (G) with minimal immunoreactivity detected in db+ mice (E). In LDRG of db/db mice, many CD68-positve macrophages were detected but not in db+ LDRG (compare K and I, arrows). COX-2 expression was also detected in both small and large neuronal populations (O, arrowhead and arrow respectively) in db/db but not db+ mice. SB 203580 treatment did not change the patterns of immunoreactivity of iNOS, TNF-α, CD68, and COX2 in db+ mice (compare A to B, E to F, I to J, and M to N). In comparison, SB203580 treatment significantly decreased the immunoreactivity of iNOS, TNF-α, CD68 and COX2 in LDRG of db/db mice (compare C to D, G to H, K to L, and O to P). N = 4, Bar = 30 μm.

To determine the cellular localization of the p38-dependent inflammatory mediators in LDRG, immunohistochemistry studies were performed for iNOS (Fig. 6A, B, C, D), TNF-α (Fig. 6E, F, G, H), CD68 (a macrophage marker, Fig. 6I, 6J, 6K, 6L), and COX2 (Fig. 6M, N, O, P). Both db+ and db/db mice were treated with vehicle or vehicle containing SB203580 intrathecally for 1 wk. Enhanced immunoreactivity for iNOS, TNF-α and COX2 was detected in LDRG of db/db mice when compared to LDRG of db+ mice. The immunopositive cells for iNOS, TNF-α, and COX2 are mostly neurons, and include both small- to medium- and large sizes (Fig. 6C, G, K, O). In addition, increased numbers of CD68-positive macrophages were detected in db/db mouse LDRG, suggesting the involvement of cell-mediated inflammation (Fig. 6K). SB203580 treatment significantly reduced the immunoreactivity of iNOS, TNF-α, CD68, and COX2, in DRG neurons of db/db mice (compare Fig. 6C to 6D, G to 6H, K to 6L, and 6O to 6P).

**Figure 7:**
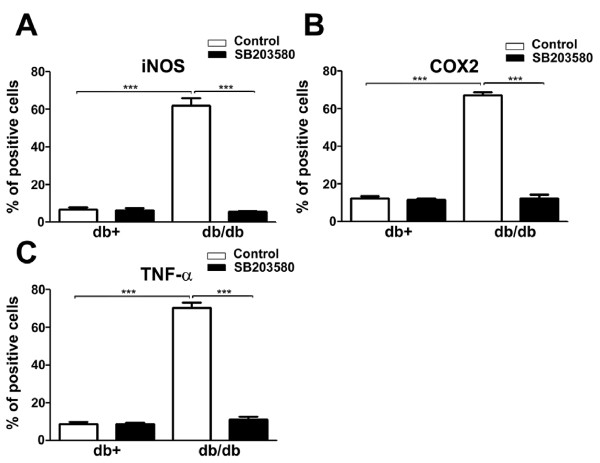
**SB203580 treatment decreases the expression of iNOS, COX2, and TNF-α in db/db LDRG neurons**. The percentages of immunopositive LDRG neurons for iNOS (A), COX2 (B), and TNF-α (C) were measured in db+ and db/db mice. The mice were treated with vehicle control (artificial CSF containing 10% DMSO) or vehicle with SB203580. Significant increases in the percentages for iNOS, COX2, and TNF-α were detected in vehicle-treated db/db mice, in comparison to db+ mice. SB203580 treatment significantly blocks the increased percentages of iNOS, COX2, and TNF-α in db/db mice to levels comparable with the nondiabetic control (db+) mice. N = 4, ***p < 0.001.

We further quantified the numbers of immunopositive LDRG neurons of db+ and db/db mice treated with vehicle control and SB203580 for iNOS (Fig. 7A), COX2 (Fig. 7B), and TNF-α (Fig. 7C). Increased percentages of neurons positive for all three molecules in db/db mice were observed in comparison to db+. In db/db mice, SB203580 significantly decreased the percentages of COX2, iNOS, and TNF-α positive LDRG neurons.

**Figure 8:**
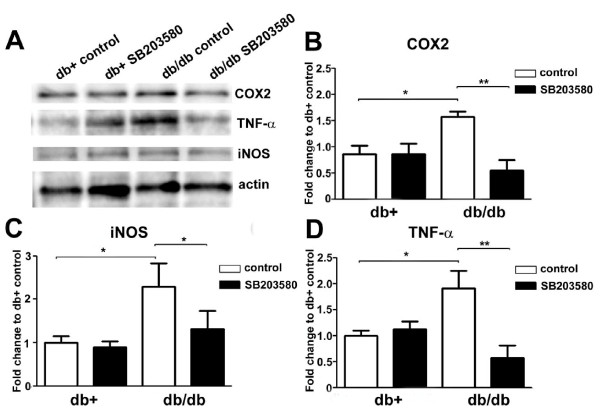
**SB203580 treatment inhibits protein expression of inflammatory mediators in db/db mice**. A: Representative immunoblots of LDRG from db+ and db/db mice. After 1 wk treatments with vehicle control or vehicle with SB203580, immunoblots were performed for iNOS, COX2, and TNF-α. Increased protein levels for all three inflammatory mediators were demonstrated in LDRG of db/db mice compared to db+ littermates. This upregulation was inhibited by treatment with SB203580. Actin served as a loading control. B, C, D: Densitometric analysis of COX2 (B), iNOS (C), and TNF-α (D) demonstrated significant upregulation of each respective protein in db/db mice that is inhibited by SB203580. N = 4, *p < 0.05, **p < 0.01.

Immunoblots of COX2, iNOS, and TNF-α (Fig. 8A) were performed in order to quantify the levels of these inflammatory proteins. Densitometric analysis confirmed that the levels of COX2 (Fig. 8B), iNOS (Fig. 8C) and TNF-α (Fig. 8D) were increased in db/db mice in comparison to levels detected in db+ mice. SB203580 treatment inhibited this upregulation of inflammatory protein expression. However, in contrast to what is observed in db/db mice, SB203580 treatment did not have an effect on the protein levels of db+ mice (Fig. 8).

## Discussion

P38 has been reported to mediate many types of pain [7]. Here we are the first to report a role for this protein kinase in mechanical allodynia associated with PDN in an animal model of type 2 diabetes. Previously, we reported that NGF-Trk A signaling is elevated in db/db mice [15]. The current study focuses on the mechanism underlying this role of p38 kinase in PDN and demonstrates that p38 is phosphorylated via NGF signaling in DRG neurons in the type 2 diabetic model. In turn, NGF-mediated p38 phosphorylation leads to mechanical allodynia in the db/db mouse by upregulation of multiple inflammatory mediators in LDRG.

Our results demonstrated the phosphorylation of p38 in DRG of db/db mice at the early stage of mechanical allodynia. P38 phosphorylation was transient (from 5-10 wk) and in parallel with the increased NGF expression in DRG and the development of mechanical allodynia [15]. The cause of this p38 phosphorylation is unclear, although the effect appears to result from exposure to hyperglycemia. We previously found that p38 is activated in neurons in culture in high glucose conditions [18]. Phosphorylation of p38 is also reported in other tissues of db/db mice [19]. Adhikary and colleagues detected p38 activation in kidneys of mouse models of type 1 and type 2 (db/db) diabetes, as well as kidneys of diabetic patients. Levels of pp38 in kidneys increased (2-6 fold) following the onset of diabetes in interstitial macrophages and myofibroblasts of db/db mice in a manner that was associated with increased NGF expression downstream of hyperglycemia, and increased HbA(1)c. Our results demonstrate a comparable time course of p38 phosphorylation in DRG, suggesting similar mechanisms could be involved in the neuronal systems. In the current study, the p38 phosphorylation in DRG of db/db mice returned to the control levels at 12 wk of age. This reduction is likely a result from lack of NGF support at this stage [15]. Future studies using anti-diabetic agents will elucidate the role of hyperglycemia in the phosphorylation of p38 in DRG of db/db mice.

Our findings of p38 phosphorylation parallel reports in a variety of inflammatory painful conditions, including osteoarthritic pain [20], bone pain [21], and complete Freund's adjuvant-induced inflammatory pain [22]. As predicted from our data, p38 is also involved in many neuropathic painful conditions, including nerve injury [13,23], neuropathic pain from type 1 diabetes [24], and central pain syndromes [25]. In the peripheral nervous system, p38 is activated in primary sensory DRG neurons by noxious stimuli [26], inflammatory pain [10,27] and nerve injuries [28,29].

In the current study, we found that p38 is phosphorylated in small- to medium- sized LDRG neurons during the period of mechanical allodynia in db/db mice. Our findings are consistent with those of the STZ model of type 1 diabetic pain [14,24], Obata and colleagues reported that most pp38 immunoreactivity is detected in small to medium-sized neurons, which also corresponds with our findings. [26,29]. In the spinal cord of similar pain models, pp38 immunoreactivity is detected in microglia, secondary sensory neurons, and astrocytes [7]

We demonstrate that NGF is an important factor to induce p38 activation in db/db mice. Anti-NGF treatment completely blocked p38 phosphorylation in LDRG in db/db mice and subsequent mechanical allodynia. NGF activates p38 in primary DRG neurons [10,30]. It has been proposed that NGF-activated p38 could increase the expression of transient receptor potential vallinoid (TRPV) 1 receptor [10] and transient receptor potential subfamily A 1 receptor [30] to mediate thermal and cold hyperalgesia respectively. According to Puntambekar and Mukherjea, the p38-mediated TRPV1 upregulation is secondary to activation of the Trk A receptor and its downstream RAC1/NADPH oxidase pathway [31]. The consistent time course shared by p38 phosphorylation, NGF upregulation, and Trk A phosphorylation in LDRG strongly suggests that the NGF-Trk A/p38 pathway mediates mechanical allodynia in db/db mice [15].

In the current study, we demonstrate that intrathecal administration of SB203580 completely blocks p38 phosphorylation and mechanical allodynia. Intrathecal administration of SB203580 blocks p38 phosphorylation in both DRG neurons and spinal cord cells (including neurons, astrocytes and microglia), according to previous studies [13,14,32,33]. In addition, other p38 inhibitors are used with the same result [24]. One caveat to our approach and that of our colleagues is that intrathecal administration of p38 inhibitors prevents p38 activation in both central and peripheral areas of the nervous system, thus the roles of site-specific p38 activation cannot be determined using this approach. As a result, we cannot eliminate the hypothesis that inhibitory effects of SB203580 on the development of PDN could result from effects in the spinal cord. Regardless, our findings support the use of p38 inhibitors for treating PDN of type 2 diabetes. Future studies should address the distinct roles of central versus peripheral p38 activation in PDN of type 2 diabetes.

We demonstrate that p38-mediated mechanical allodynia in db/db mice is primarily mediated by small to medium-sized LDRG neurons that are immunopositive for SP. We previously reported that these SP-positive neurons are NGF-positive neurons with Trk A receptors [15]. Thus, our current results support the current hypothesis that NGF-dependent neurons are primarily responsible for the development of allodynia. In contrast, the numbers of IB4-labelled neurons are not affected in db/db mice, suggesting that GDNF dependent neurons do not play a role in mediating mechanical allodynia in db/db mice. These results are consistent with our previous findings that GDNF expression and the percentages of IB4-labelled neurons do not change in db/db mice [15]. In parallel with our findings, NGF, but not GDNF-sensitive neurons, mediate discogenic pain [34]. According to Ramer and Bradbury, intrathecal injection of NGF treatment induces more extensive expression of the P2X3 receptor than GDNF in both DRG and spinal cord dorsal horn, and leads to chronic pain [35]. The upregulation of SP-positive neurons in our current study is reduced by SB203580, supporting our contention that p38 mediates SP-upregulation in the DRG of db/db mice. There are two potential signaling mechanisms that may underlie this effect: 1) NGF may directly induce Trk A-dependent p38 phosphorylation, or 2) NGF could indirectly activate interleukin 1-beta-dependent p38 activation to promote SP expression [36]. We are currently performing experiments to distinguish between these two possibilities.

Our findings suggest that there are inflammatory reactions that occur in DRG of type 2 diabetes. Specifically, we detected increasing macrophage infiltration as well as upregulation of multiple inflammatory mediators in LDRG of db/db mice. Joachim and colleagues determined that NGF, a proinflammatory cytokine, induces inflammation in multiple tissues, including skin [37]. It is known that NGF administration to skin not only induces an inflammatory reaction but also increases the number of dermal SP and CGRP-positive nerve fibers. Previously, we have reported that there is increased NGF expression in dermal inflammatory cells and nerve fibers in db/db mice, indicating that similar NGF-mediated inflammation occurs in the skin of mice with type 2 diabetes [15]. Our current study provides further evidence that similar inflammation occurs in DRG of db/db mice.

Our study demonstrated an upregulation of COX2 expression in DRG of db/db mice. Kellogg et al reported increased COX2 expression in DRG of STZ rats, a model of type 1 diabetes and suggested potential roles of COX2 in the mediation of diabetic neuropathy, which is consistent with our findings [38]. COX2 also mediates other diabetic complications, including nephropathy [39]. In order to study COX2, Bujaslska et al used specific COX2 inhibitors to demonstrate that COX2 mediates hyperalgesia in the STZ model of type 1 diabetes. [40]. Our study demonstrates that COX2 expression is mediated by p38 in the DRG of db/db mice. In support of our findings, Amaya and colleagues reported a p38-dependent COX2 expression by IL-1 [27]. In contrast, Kitazawa et al demonstrated that C-peptide-induced COX2 upregulation is dependent on the PKC/IkappaB/NF-kappaB signaling pathway in fibroblasts [41]. We acknowledge that there are multiple signaling mechanisms to regulate COX2 expression and at this time we can not exclude the PKC/IkappaB/NF-kappaB signaling pathway, which could be an upstream or downstream mechanism of p38 activation to increase COX2 expression.

We detected increased iNOS expression in DRG of db/db mice and suggest this upregulation could be a mechanism of mechanical allodynia. In agreement with our hypothesis, iNOS knockout mice have increased resistance to diabetic neuropathic complications, including impaired nerve conduction velocities and small fiber sensory neuropathy, indicating that iNOS could be an important mediator of PDN [42]. There is evidence to imply that diabetes-induced increased NO levels result from cellular signaling via advanced glycation and lipoxidation end products (AGEs/ALEs) [43]. The increased NO levels likely contribute to tactile hyperalgesia in the STZ model of type 1 diabetes [44]. In support of our findings in type 2 diabetes, Bujalska et al reported that intrathecal administration of a specific inhibitor of iNOS, but not neuronal NOS, prevents hyperalgesia in STZ-induced type 1 diabetes [40].

We report that there is a p38-dependent TNF-α upregulation in DRG of db/db mice. Sherry et al demonstrate that p38 also mediates augmented lipopolysaccharide induced TNF-α expression in peritoneal macrophages of db/db mice [45]. In contrast to their findings, we detected increased DRG expression of TNF-α in neurons instead of in infiltrating macrophages. However, macrophages could contribute to both inflammation and pain by increasing interleukins in DRG, including, IL-6 [46], and IL-12 [47]. Our findings strongly suggest that NGF increases TNF-α expression via p38. However, other NGF-dependant signaling mechanisms have been reported to enhance TNF-α expression, including NF-kappaB. In support of our results, neuronal-derived TNF-α expression is upregulated in DRG of nerve injury models [48,49]. One way that TNF-α could increase mechanical nociception is by activating p38 via the TNF receptor 1 to modulate the tetrodotoxin-resistant sodium channel in DRG [50]. This molecular mechanism could also occur in our model with elevated neuronal p38-mediated-TNF-α expression which, in turn, could serve as an autocrine factor to cause secondary p38 activation via the cell surface TNF receptor 1. The gene expression of TNF-α was decreased by SB203580 in db+ mice. In contrast, SB203580 did not alter COX2 or iNOS RNAs. The finding suggests that there is a baseline p38-mediated regulation of TNF-α expression. Baseline p38-mediated TNF-α may be regulated by the trivial NGF level in LDRG of control mice or via other pathways like IL-β inhibition of G protein-coupled receptor kinase 2 (GRK2) [51].

In summary, the current study demonstrates the phosphorylation of p38 and the upregulation of multiple inflammatory mediators including COX2, iNOS, and TNF-α in DRG neurons of db/db mice. Our data suggest that inflammation in DRG could mediate mechanical allodynia in type 2 diabetes. While current guidelines for treating PDN only use neuropathic pain regimens [52], our results suggest that a combination of both neuropathic and anti-inflammatory therapies that target COX2, iNOS, and TNF-α will improve the current standard treatment for PDN of type 2 diabetes. Since p38 mediates multiple inflammatory mediators in PDN of type 2 diabetes, clinical studies using p38 inhibitors could potentially provide a better approach than using multiple inhibitors for mediators downstream of this mechanism to alleviate PDN of type 2 diabetes [53].

## Methods

### Animals

Male C57BLKS db/db mice were purchased from Jackson Laboratories (Bar Harbor, Maine; stock number 000662). The homozygous (*Lepr^db^*/* Lepr^db^*, or db/db) mice were used as a model of type 2 diabetes, while heterozygous mice (*Lepr^db^*/+, or db+) served as nondiabetic controls. Analyses and procedures were performed in compliance with protocols established by the Animal Models of Diabetic Complications Consortium (AMDCC) http://www.amdcc.org and were approved by the Use and Care of Animals Committee at the University of Michigan. All possible efforts were made to minimize the animals' suffering and the number of animals used.

### Mechanical Allodynia

The animals were placed in a Plexiglas cage with mesh flooring and allowed to acclimate for 1 h. A logarithmic series of calibrated monofilaments (Von Frey hairs; Stoelting, Wood Dale, IL) with bending forces from 1 to 10 g were applied to the mid-plantar surface of the hind paw and pressed to the point of bending. Brisk withdrawal of the stimulated paw was recorded as a positive response. Testing began with the 1 g filament, followed by larger filaments if no response was observed, using the up-down method [54], with a 10 min. interval to allow the animals to recover between tests. Although all responses were noted, counting of the critical 6 data points did not begin until the response threshold was first crossed. The resulting pattern of the 6 positive and negative responses was tabulated, and the 50% gram threshold was calculated using the formula described previously [55]. Mechanical allodynia was determined by a significant decrease in the mechanical threshold of the db/db mouse in comparison to that of a db+ littermate.

### Real time RT-PCR

Total RNA was extracted from L4-6 DRG (LDRG) using the RNeasy Kit (Qiagen, Valencia, CA) according to the manufacturer's instructions. Six DRG (bilateral L4-6) were used for each animal and a total of 4 animals were used per condition. Reverse transcription was performed using the iScript cDNA Synthesis Kit (BioRad, Hercules, CA). Briefly, 5× iScript Reaction Mix, 1 μl iScript Reverse Transcriptase and total RNA template were added to a final volume of 20 μl. Reaction conditions were 5 min. at 25°C, 30 min. at 42°C and 5 min. at 85°C. PCR was performed as described previously [56] using the primer sequences: COX2: sense 5'-GAT GAG CAA CTA TTC CAA ACC AG, antisense 5'-CCG CTC AGG TGT TGC ACG TAG; iNOS sense 5'-GGG CAG CCT GTG AGA CCT T, antisense 5'-TGA GGG CTC TGT TGA GGT CTA; TNF-α sense 5'-AGC CGA TTT GCT ATC TCA TAC CAG, antisense 5'-CCT TCA CAG AGC AAT GAC TCC A; GAPDH sense 5'-TCC ATG ACA ACT TTG GCA TCG TGG-3', antisense 5'-GTT GCT GTT GAA GTC ACA GGA GAC-3'.

All real-time PCR reactions were carried out in 96-well PCR plates sealed with iCycler Optical Sealing Tape (BioRad). The PCR reactions contained 1× SYBR Green iCycler iQ mixture (BioRad), 0.2 μM of each forward and reverse primer, and cDNA preparation to 25 μl total volume. The PCR amplification profile was 94°C for 2 min, 35 cycles of denaturation at 94°C for 30 s, annealing at 60°C for 1 min, and extension at 72°C for 30 s, followed by 72°C for 5 min. The mRNA expression levels of the genes were tested, and amplification and fluorescence detection were performed using iCycler iQ Real-time Detection System (BioRad). At the end of the PCR, melting curves were obtained from 46 subsequent temperature increments by measuring fluorescence every 10 s with + 0.5°C/step increment, beginning at 72°C. The quality of PCR products was determined by melting curve analysis. The fluorescence threshold value was calculated by the iCycler iQ system software, and the levels were normalized to values obtained for GAPDH. A non-template control [57] was run with every assay.

### Immunoblots

Following deep anesthesia, L4-6 DRG were dissected from 4 mice per condition (db/db and db+) and homogenized in ice-cold T-PER Tissue Protein Extraction Reagent (Pierce Biotechnology, Rockford, IL) containing protease inhibitors (1 μM sodium orthovanadate and 1 μM sodium fluoride; Sigma Life Science, St. Louis, MO). Lysates were sonicated for 5 s, centrifuged and processed for protein concentration using D_C _Protein Assay Reagents (BioRad). 50 μg of protein were boiled in 2× sample buffer, separated on a SDS-PAGE gel, and transferred to a PVDF membrane. Membranes were blocked and incubated overnight at 4°C with primary antibodies: phospho-p38 (pp38, 1:1000, rabbit polyclonal, Cell Signaling, Danvers, MA), total p38 (1:1000, rabbit polyclonal, Cell Signaling Danvers, MA), COX2 (1:1000, Cayman Chemical, Ann Arbor, MI), iNOS (1:1000, Abcam Inc, Cambridge, MA), and TNF-α (1:1000, Abcam, Cambridge, MA). Membranes were then rinsed and incubated with HRP-conjugated secondary antibodies for 1 h at 25°C and processed with chemiluminescence substrate (Thermo Fisher Scientific, Rockford, IL) before being exposed to Hyperfilm (Amersham, Piscataway, NJ). Densitometry was performed using Image J software, and the results were normalized against actin densities from the same sample.

### Immunohistochemistry

Four mice from each group (db+ and db/db) were deeply anesthetized and perfused with 2% paraformaldehyde in phosphate buffered saline (PBS, pH 7.2, 0.1 M). L4-L6 DRG were dissected and post-fixed by immersion in 2% paraformaldehyde overnight at 4°C, then rinsed in graded sucrose solutions (5-30% in PBS), embedded in mounting media (OCT), and flash-frozen in liquid nitrogen. Tissue sections (10 μm) were cut and mounted onto SuperFrost Plus slides (Fisher Scientific, Pittsburgh, PA) and stored at -80°C until ready for use. For immunolocalization, tissue sections were thawed on a warming plate (55°C for 10 min), hydrated with PBS, and blocked in 0.1% TX-100 and 5% non-fat dry milk in PBS. Sections were then incubated at room temperature for 16-24 h with primary antibodies: pp38 (1:1000), SP (Rat monoclonal, 1:500, Abcam), iNOS (1:1000), TNF-α (1:1000), or COX2 (1:1000). Sections were then rinsed 3 times in PBS and incubated with secondary antiserum conjugated with different fluorophores (AlexaFluor 488, 594, or 647, Invitrogen, Carlsbad, CA). Neurons were identified using NeuN antibody (1:250, Neuronal Nuclei, Alexa Fluor 488, Millipore, Temecula, CA). For IB4 labeling studies, the sections were incubated with AlexaFluor 594 labeled *Griffonia simplicifolia *Isolectin B-4 (1:500, Invitrogen) with 5% non-fat dry milk in PBS for 1 h. Sections were rinsed and mounted with ProLong^® ^Gold antifade reagent (Invitrogen). To ensure specificity, sections were incubated with primary or secondary antisera alone to confirm there were no nonspecific immunoreactions. Fluorescent signals were examined using an Olympus FluoView 500 laser scanning confocal microscope.

The percentage of immunopositive cells was analyzed by counting the number of immunopositive neurons and multiplying by (100/total number of neurons). Cell size distribution studies were performed on the same image. A total of 6 LDRG from each animal were measured. Images of LDRG sections were captured with a Nikon camera (Nikon Microphoto-FXA), and the number of immunoreactive neuronal profiles was counted in a blinded fashion. Every tenth section was picked from a series of consecutive LDRG sections (10 μm), and three to four sections were counted for each LDRG and expressed as the percentage of total neuronal profile measured by NeuN immunohistochemistry [10]. Sections were incubated with primary antisera alone, or secondary antisera alone, to ensure specificity. No significant nonspecific immunolabeling was detected in either control condition.

### Anti-NGF treatment

To inhibit NGF action during the period of allodynia, we administered anti-NGF (10 mg/kg, mouse monoclonal antibody clone AS21, Exalpha Biologicals, Maynard, MA) or control IgG, intraperitoneally, once weekly to db+ and db/db mice at 6 and 7 wk of age for 2 wk [15,58]. L4-6 LDRG were collected at 8 wk of age and processed for pp38/p38 immunoblots.

### Minipump placement

An osmotic minipump (Alzet minipump model 1007D, Duent Corporation, Cupertino, CA) was used for continuous intrathecal infusion into the lumbar spinal cord region. The 100 μl volume minipump is designed with a 0.51 μl/hr infusion rate. The minipumps were filled with artificial cerebrospinal fluid (CSF) with 10% dimethyl sulfoxide (DMSO) with or without SB203580 (1 mg/ml, EMD Chemicals, Gibbstown, NJ). The minipumps were implanted into the dorsal subcutaneous space between the shoulder blades of each mouse at 7 wk of age under sterile conditions. A caudally directed polyethylene cannula (Becton Dickinson and Company, Sparks, MD) was threaded subcutaneously at the level of the L5 spinal process. The L5 spinal process was removed and the tip of the cannula was then inserted into the subarachnoid space at the L5 level. The intrathecal infusion lasted for 1 wk at which time the mice reached 8 wk of age. Behavior studies were performed, followed by tissue collection.

### Data presentation and statistical analyses

All data are presented as group means ± SEM. The data between db+ and db/db mice of the same age were analyzed using the Mann-Whitney test. Statistical comparisons between different age groups were made by one-way ANOVA tests followed by a post hoc Tukey's multiple comparison test. A p-value of less than 0.05 was considered statistically significant.

## Competing interests

The authors declare that they have no competing interests.

## Authors' contributions

HTC, JRD, JMH, SSO, and YH carried out the studies described. HTC and ELF contributed to designing the present study and analyzing the results. HTC, JRD, and ELF wrote the manuscript. All authors read and approved the final manuscript.
